# IGLC3^-^ tumor cells drive chemoresistance in colorectal cancer by polarizing SPP1^+^ macrophages via the CD44-Wnt-BTF3 axis

**DOI:** 10.3389/fimmu.2026.1731216

**Published:** 2026-04-01

**Authors:** Feihu Yan, Yunjie Shi, Hantao Wang, Bingchen Chen, Kaiwen Sheng, Jing Wu, Wei Zhang, Hao Wang, Xu Li

**Affiliations:** Department of Colorectal Surgery, Changhai Hospital of Naval Medical University, Naval Medical University, Shanghai, China

**Keywords:** BTF3, chemoresistance, colorectal cancer, IGLC3- tumor cells, SPP1^+^ macrophages

## Abstract

Colorectal cancer (CRC) remains a leading cause of cancer-related mortality worldwide, with tumor heterogeneity and chemoresistance posing significant therapeutic challenges. In this study, we investigated the role of tumor-macrophage interactions in CRC progression. Using single-cell RNA sequencing (scRNA-seq) analysis from public database, patient-derived organoid models, and *in vivo* mouse models, we demonstrated that IGLC3^-^ tumor cells secreted TGF-β to polarize M0 macrophages into an SPP1^+^, M2-like phenotype. These SPP1^+^ macrophages enhanced tumor cell proliferation, stemness, and migration via CD44–Wnt–BTF3 signaling pathway. Inhibition of CD44 or Wnt signaling with HH1 or a Wnt inhibitor effectively reversed macrophage-mediated chemoresistance and suppressed tumor growth and metastasis. Notably, HH1 exhibited superior safety compared to the Wnt agonist, making it a promising candidate for combination therapy. These findings provide novel insights into tumor heterogeneity and macrophage-mediated chemoresistance, highlighting actionable targets within the tumor microenvironment to improve CRC treatment outcomes.

## Graphical Abstract

**Figure:**
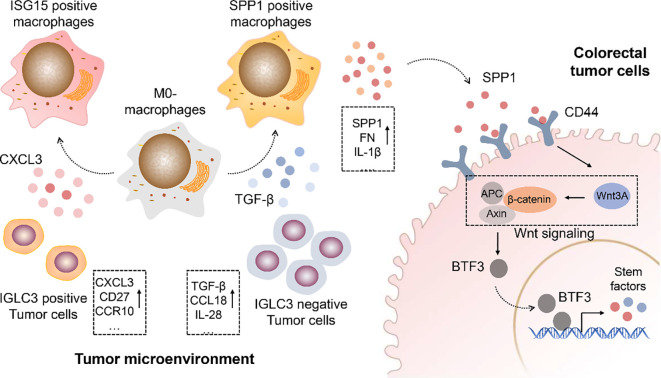
Schematic illustration of the proposed mechanism by which IGLC3-defined tumor cell subsets regulate macrophage polarization and drive chemoresistance in colorectal cancer.

## 1 Introduction

Colorectal cancer (CRC) remains a leading cause of cancer-related mortality worldwide, primarily due to its pronounced tumor heterogeneity and frequent development of therapy resistance (1, 2). This heterogeneity encompasses genetic, epigenetic, and phenotypic variations within tumor cell populations (3, 4), giving rise to subpopulations with distinct biological behaviors. For example, tumor cells expressing CD133 or CD44 often exhibit enhanced proliferative capacity, stemness, and resistance to treatment (5–7). A comprehensive understanding of these subgroups is essential for elucidating the mechanisms driving CRC progression and therapeutic failure.

The tumor microenvironment (TME) plays a central role in shaping CRC behavior and therapeutic outcomes (8, 9). Among the diverse stromal and immune components within the TME, tumor-associated macrophages (TAMs) are particularly influential. TAMs exhibit remarkable plasticity and can adopt either a pro-inflammatory M1-like phenotype or an anti-inflammatory, pro-tumorigenic M2-like phenotype. M2-like TAMs contribute to tumor progression, immune suppression, and metastasis through cytokine production, growth factor signaling, and direct cell–cell interactions (10–12). SPP1 (osteopontin)-expressing macrophages have recently emerged as a functionally distinct subset of TAMs associated with tumor progression. SPP1, a multifunctional glycoprotein involved in cell signaling and extracellular matrix remodeling, contributes to immune modulation and cancer development (13). Elevated SPP1 expression correlates with poor prognosis and therapy resistance in several cancers, including CRC (14, 15). However, the specific correlation between SPP1^+^ macrophages and tumor cell subtypes remains inadequately understood.

Single-cell RNA sequencing (scRNA-seq) offers a powerful platform to resolve the cellular heterogeneity of tumor and immune compartments, revealing previously unrecognized interactions within the TME. Accumulating evidence suggests that TAMs can enhance cancer stem cell (CSC) properties, thereby maintaining tumor plasticity and fueling resistance (16, 17). In this study, we aimed to investigate how specific CRC tumor cell subtypes interact with macrophages to influence tumor behavior. Using scRNA-seq analysis from public database, patient-derived organoid models, and *in vivo* mouse models, we identified IGLC3^-^ tumor cells as potent inducers of SPP1^+^ macrophage polarization. We further explored how these macrophages promote tumor stemness, migration, and drug resistance through the CD44–Wnt–BTF3 signaling pathway. Finally, we assessed the therapeutic potential of targeting this axis to reverse chemoresistance and improve CRC treatment outcomes.

## 2 Materials and methods

### 2.1 Cell culture and reagents

Human CRC cell lines HCT116, SW480, along with the murine CRC cell line CT26, were obtained from the American Type Culture Collection (ATCC, USA). HCT116 and SW480 cells were cultured in RPMI-1640 medium (Gibco, USA) supplemented with 10% fetal bovine serum (FBS; Gibco, USA), while CT26 cells were cultured in Dulbecco’s Modified Eagle Medium (DMEM; Gibco, USA) with the same supplements. All cell lines were incubated at 37 °C in a humidified atmosphere with 5% CO_2_. Cell line authentication was performed using short tandem repeat (STR) profiling within six months of use. The following reagents were used: recombinant human TGF-β, CXCL3, fibronectin (FN), SPP1, 5-fluorouracil (5-Fu), and oxaliplatin (Oxa) (all from R&D Systems, USA). For macrophage depletion experiments, clodronate liposomes were obtained from Liposoma BV (Amsterdam, Netherlands) and used according to the manufacturer’s instructions. Control liposomes were applied in parallel experiments where indicated (200 μL per mouse). Cetuximab and panitumumab were obtained from Selleck (USA). The CD44 inhibitor HH1 and Wnt inhibitor (Wnt/β-catenin-IN-6) were obtained from MedChemExpress (USA).

### 2.2 Clinical specimens

CRC specimens were obtained from patients undergoing surgical resection at Changhai Hospital, with approval from the Institutional Review Board. All patients provided written informed consent prior to sample collection. Forty-four tumor specimens were classified according to the American Joint Committee on Cancer (AJCC) 8th edition staging system into high-stage (III–IV) and low-stage (I–II) groups, and metastatic (M1) and non-metastatic (M0) groups. Another set of 31 tumor samples was classified into responding (complete and partial response) and non-responding groups, based on the Response Evaluation Criteria in Solid Tumors (RECIST). The details of patient information were shown in **Supplementary Table 1** (supplementary information). Correlation analysis between immune cell infiltration and the genes IGLC3/BTF3/SLC7A5/TNS4 in CRC patients was performed using the online Sanger tool (http://sangerbox.com/home.html).

### 2.3 Macrophage-tumor cell co-culture

THP-1 human monocytes (ATCC, USA) were differentiated into M0 macrophages by treatment with 50 ng/mL phorbol 12-myristate 13-acetate (PMA; Sigma-Aldrich) for 24 hours. After PMA removal, cells were incubated in complete medium without PMA for an additional 24 hours to stabilize differentiation. M1 macrophages were generated by stimulating M0 macrophages with 20 ng/mL interferon-gamma (IFN-γ; PeproTech, USA) and 100 ng/mL lipopolysaccharide (LPS; Sigma-Aldrich, USA) for 24 hours. M2 macrophages were induced by treating M0 macrophages with 20 ng/mL interleukin-4 (IL-4) and 20 ng/mL interleukin-13 (IL-13; PeproTech, USA) for 24 hours. SPP1^+^ and SPP1^-^ macrophages were isolated from freshly dissociated tumor tissues by flow cytometry using antibodies against SPP1 (ab216402, Abcam, UK) and CD68 (11-0689-42, Thermo Fisher Scientific, USA). For co-culture experiments, differentiated macrophages were seeded into Transwell inserts (0.4 μm pore size, Corning), while tumor cells—either IGLC3-overexpressing or vector-transduced HCT116/SW480 cells—or patient-derived organoids embedded in Matrigel were plated in the lower chamber at a tumor-to-macrophage ratio of 1:10. After 72 hours of co-culture, macrophages or tumor cells were harvested for subsequent analysis.

### 2.4 Single-cell RNA sequencing analysis

Single-cell RNA sequencing data were obtained from the NCBI GEO database (accession number GSE166555), which includes 25 samples derived from 12 CRC patients—13 tumor samples and 12 adjacent normal tissue samples. The data were analyzed using the Seurat package (v4.0) in R. Raw sequencing reads were quality-filtered, and cells with fewer than 200 or more than 2,500 unique gene counts were excluded. Dimensionality reduction was performed using Uniform Manifold Approximation and Projection (UMAP), and clustering was conducted with the FindClusters function (resolution = 0.5). Marker genes for each cluster were identified using the FindMarkers function, with adjusted p-values <0.05 considered significant. Cell subtypes were annotated based on established gene expression profiles. Detailed information about the single-cell analysis is provided in the **Supplementary Methods**.

### 2.5 Animal studies

All animal procedures were approved by the Institutional Animal Care and Use Committee of Changhai Hospital. Six-week-old BALB/c and NOD-SCID mice were subcutaneously injected with 1 × 10^6^ CT26 cells. For macrophage depletion, BALB/c mice received intraperitoneal injections of 200 μL clodronate-liposomes (Liposoma, Netherlands) or control PBS-liposomes on days 2, 7, and 12 after tumor implantation to assess the role of SPP1^+^ macrophages, CT26 cells were co-cultured *in vitro* with SPP1^+^ or SPP1^-^ macrophages isolated from tumor-bearing mice. These preconditioned CT26 cells were then injected subcutaneously into BALB/c mice. On days 5 and 10 post-implantation, mice received intratumoral injections of either SPP1^+^ or SPP1^-^ macrophages. Therapeutic interventions—including oxaliplatin (Oxa, 5 mg/kg), Wnt inhibitor (2.5 mg/kg), HH1 (2.5 mg/kg), or their combinations—were administered via tail vein injections on days 12, 15, and 18. Tumor dimensions were measured with digital calipers, and tumor volume was calculated using the formula: Volume = (length × width²)/2.

### 2.6 Statistical analysis

All statistical analyses were performed using GraphPad Prism 6 (USA). Data are presented as the mean ± standard deviation. The statistical significance of differences between two groups was evaluated using an unpaired two-tailed Student’s t-test. For comparisons involving three or more groups, one-way analysis of variance (ANOVA) followed by Tukey’s *post hoc* test was used to assess statistical significance. Kaplan-Meier survival curves were generated to assess the association between patient survival and clinical or molecular factors. The relationship between gene expression levels and patient survival was analyzed using Spearman’s correlation coefficient. Statistical significance was considered when p < 0.05.

## 3 Results

### 3.1 Identification of IGLC3 tumor cell subtype in colorectal cancer

**Figure 1:**
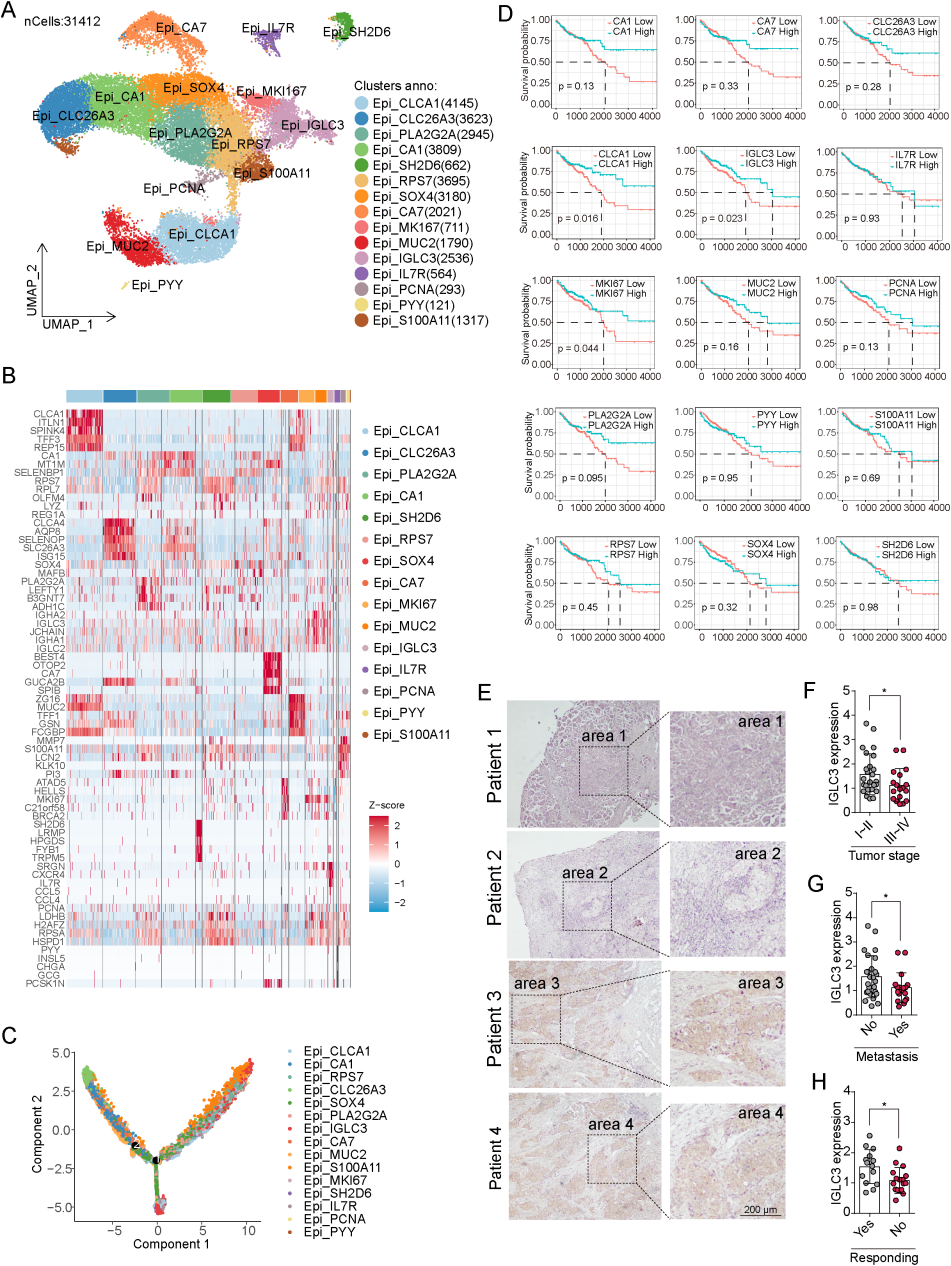
Identification of IGLC3 tumor cell subtype in colorectal cancer. **(A)** TSNE of 15 tumor cell clusters in scRNA-seq of colorectal tumor tissues, including CLCA1^+^, CLC26A3^+^, PLA2G2A^+^, CA1^+^, SH2D6^+^, RPS7^+^, SOX4^+^, CA7^+^, MKI167^+^, MUC2^+^, IGLC3^+^, IL7^+^, PCNA^+^, PYY^+^, and S100A11^+^ cell subtypes. **(B)** Heatmap of top differential gene expression between each tumor subtype. **(C)** Pseudotime trajectory of epithelial tumor cells constructed using Monocle2. The trajectory origin (leftmost region) was manually defined based on biological context. Cells are colored by pseudotime (from early to late states) to illustrate the progression from differentiated to stem-like tumor states. **(D)** Survival analysis was performed according to the infiltration score of each tumor subtype in TCGA-COAD. **(E)** Immunofluorescence staining of IGLC3 in patient-derived colorectal tumor tissues. **(F-H)** The IGLC3 expression intensity was compared between patients with stage I~II (low stage) or stage III~IV (high stage) **(F)**, with metastasis or not **(G)**, and responding to chemotherapy or not **(H)**. All experiments were independently repeated at least three times with consistent results. Data are presented as mean ± SD, and statistical analyses were performed using t test. *p<0.05.

Cancer cells within tumor tissues often exhibit considerable heterogeneity, playing distinct roles in tumor progression (18). To delineate functionally relevant tumor subpopulations, we performed single-cell RNA sequencing (scRNA-seq) analysis on 13 tumor and 12 adjacent normal tissue samples from CRC patients (GSE166555) (19). After quality control, a total of 31,412 epithelial-like cells were obtained and analyzed. Using Uniform Manifold Approximation and Projection (UMAP), we identified 15 distinct tumor cell subtypes, including CLCA1^+^, CLC26A3^+^, PLA2G2A^+^, CA1^+^, SH2D6^+^, RPS7^+^, SOX4+, CA7^+^, MKI167^+^, MUC2^+^, IGLC3^+^, IL7R^+^, PCNA^+^, PYY^+^, and S100A11^+^ tumor cell subtypes (**Figure 1A**; **Supplementary Figures 1A, B**), each characterized by unique gene expression patterns (**Figure 1B**). Pseudotime trajectory analysis revealed that the IGLC3^+^ and PYY^+^ subpopulations showed more defined differentiation paths compared to other subtypes (**Figure 1C**). To evaluate clinical relevance, we extracted signature genes of each tumor cell subtype and assessed their association with patient survival using the TCGA-COAD dataset. Notably, lower expression of IGLC3, CLCA1, and MKI167 correlated with poorer overall survival (**Figure 1D**). While previous studies have implicated CLCA1 and MKI67 in cell cycle regulation and proliferation (20, 21), the role of IGLC3 in cancer remains largely unexplored. To further validate the clinical significance of IGLC3, we analyzed its expression in normal and tumor tissues of patients from TCGA database. We found that IGLC3 was upregulated in most cancer types, including CRC (**Supplementary Figure 1C**). The protein expression of IGLC3 was upregulated in CRC tissues compared to normal tissues, which was validated by Atlas protein database (**Supplementary Figure 1D**). We further analyzed its expression in tumor samples from CRC patients categorized by stage (I–II vs. III–IV), metastatic status (M0 vs. M1), and chemotherapy response (responders vs. non-responders). Immunofluorescence staining showed significantly reduced IGLC3 expression in advanced-stage tumors, metastatic lesions, and non-responding tumors (**Figures 1E–H**). These findings are consistent with public database results and suggest that IGLC3^+^ tumor cells may represent a less aggressive subtype associated with improved prognosis in CRC.

### 3.2 IGLC3^-^tumor cells promote CRC progression through a macrophage dependent manner

**Figure 2:**
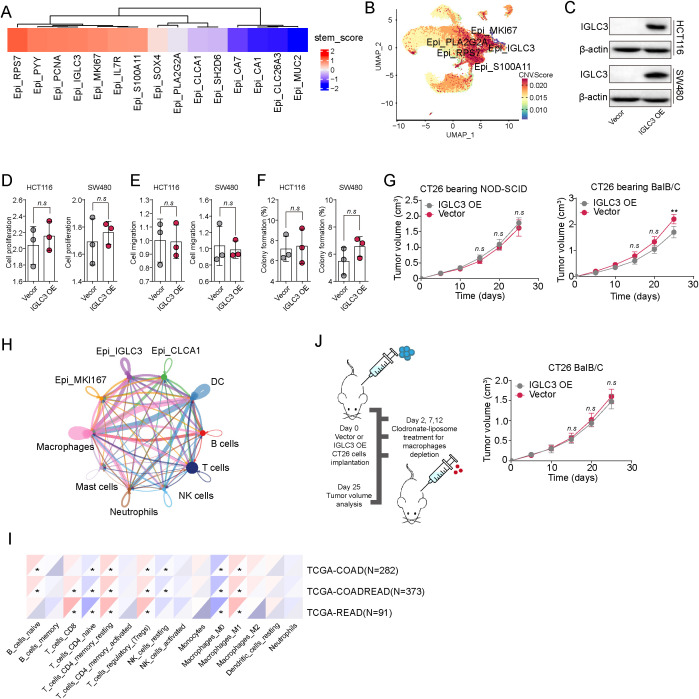
IGLC3^-^ tumor cells promote CRC progression through a macrophage dependent manner. **(A)** Heatmap of GSVA stemness gene set score of each tumors subtype. **(B)** CNV scores of each tumors subtype calculated using the infercnvpy tool. **(C)** Western blotting of IGLC3 in vector and IGLC3 overexpressed HCT116/SW480 cells. **(D-F)** Cell proliferation **(D)**, migration **(E)** and colony formation capability **(F)** of vector and IGLC3 overexpressed HCT116/SW480 cells. **(G)** Tumor volume of vector and IGLC3 overexpressed CT26 bearing BalB/C and NOD-SCID mice (n=5 in each group). **(H)** The numbers of interactions between IGLC3^+^ tumor cells and each cell subpopulation. **(I)** Correlation analysis between IGLC3 expression and each immune cell subtype in CRC patients from TCGA database, analyzed by Sanger tool (http://sangerbox.com/home.html). **(J)** Vector and IGLC3 overexpressed CT26 bearing BalB/C were treated with Clodronate-liposome on day 2, 7 and 12. Tumor volume was then determined (n=5 in each group). All experiments were independently repeated at least three times with consistent results. Data are presented as mean ± SD, and statistical analyses were performed using t test. *p<0.05, **p<0.01.

To explore the functional role of IGLC3^+^ tumor cells in CRC progression, we first performed KEGG pathway enrichment analysis on differentially expressed genes between IGLC3^+^ and IGLC3^-^ tumor cells. Surprisingly, no pathways directly related to cancer stemness or cell proliferation were significantly enriched (**Supplementary Figure 2A**). This observation led us to hypothesize that IGLC3 may not influence CRC progression by directly promoting tumor cell stemness or malignant potential. To test this, we next assessed their association with tumor cell stemness and chromosomal instability. Gene Set Variation Analysis (GSVA) revealed that IGLC3^+^ cells exhibited lower enrichment of stemness-related gene signatures compared to other subtypes (**Figure 2A**). Similarly, copy number variation (CNV) analysis using the infercnvpy tool indicated minimal chromosomal alterations in IGLC3^+^ cells (**Figure 2B**), suggesting that IGLC3 might have limited influence on malignant potential. To validate these findings functionally, we overexpressed IGLC3 in HCT116 and SW480 CRC cell lines (**Figure 2C**). Overexpression did not significantly affect cell proliferation (**Figure 2D**), migration (**Figure 2E**), or colony-forming ability (**Figure 2F**), indicating that IGLC3 does not intrinsically regulate tumor cell aggressiveness. We next hypothesized that IGLC3 may influence CRC progression through modulation of the tumor immune microenvironment. To test this, CT26 cells with or without IGLC3 overexpression were implanted into both immunodeficient NOD-SCID and immunocompetent BALB/c mice. In NOD-SCID mice, tumor growth was comparable between groups. However, in BALB/c mice, IGLC3-overexpressing tumors grew significantly slower than controls (**Figure 2G**), implicating an immune-dependent mechanism. To identify potential immune cell mediators, we performed ligand–receptor interaction analysis using scRNA-seq data and observed that IGLC3^+^ tumor cells most frequently interacted with macrophages (**Figure 2H**). Correlation analysis based on TCGA-COAD data further supported this finding: IGLC3 expression was negatively correlated with M0 macrophage infiltration and positively correlated with M1 macrophages (**Figure 2I**). To confirm that macrophages were essential for the observed IGLC3-dependent tumor suppression, we depleted macrophages in BALB/c mice using clodronate liposomes. This treatment abolished the growth-suppressive effect of IGLC3 overexpression (**Figure 2J**), demonstrating that IGLC3 exerts its tumor-suppressive effects through modulation of macrophage activity.

### 3.3 IGLC3^-^ tumor cells promote SPP1^+^ macrophage polarization

**Figure 3:**
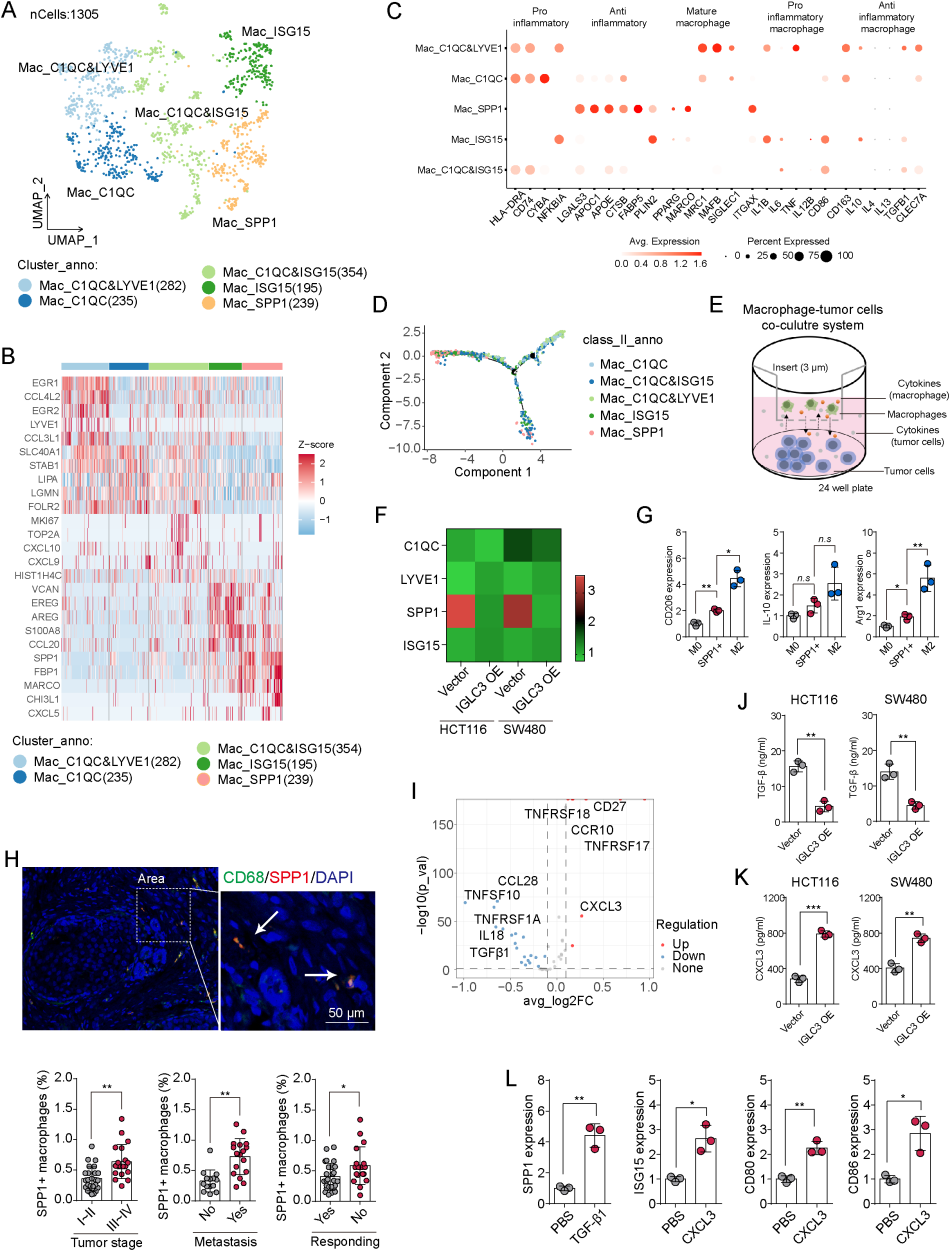
IGLC3^-^ tumor cells promote SPP1^+^ macrophage polarization. **(A)** TSNE of 5 macrophage cell clusters in scRNA-seq of colorectal tumor tissues, including C1QC&LYVE1^+^ macrophages, C1QC^+^ macrophages, C1QC&ISG15^+^ macrophages, ISG15^+^ macrophages and SPP1^+^ macrophages. **(B)** Heatmap of top differential gene expression between each macrophage subtypes. **(C)** Dot plot of inflammatory macrophages marker genes in each macrophage subtype. **(D)** The developmental trajectory between macrophage subtypes by monocle2 analysis. **(E)** Schematic diagram of macrophage and tumor cells co-culture model. **(F)** Heatmap of C1QC, LYVE1, SPP1 and ISG15 at mRNA level in THP-1 derived M0 macrophages co-cultured with vector and IGLC3 overexpressed HCT116/SW480 cells. The PBS treated THP-1 cells were set as control. **(G)** mRNA expression of CD206, IL-10 and Arg1 in patients derived SPP1^+^ macrophages (tumor tissues), and peripheral blood mononuclear cells derived M0 and M2 macrophages (blood). **(H)** Immunostaining of CD68 and SPP1 in patients derived colorectal tumor tissues. The CD68 and SPP1^+^ cells distribution was compared between patients with stage I~II (low stage) or stage III~IV (high stage), with metastasis or not, and responding to chemotherapy or not. **(I)** Volcano plot of differentially expressed cytokine-related genes between IGLC3^+^ and IGLC3^-^ tumor cells identified from our scRNA-seq dataset (GSE166555). **(J, K)** Elisa of TGF-β and CXCL3 in supernatant of vector and IGLC3 overexpressed HCT116/SW480 cells. **(L)** mRNA expression of SPP1, ISG15, CD80 and CD68 in THP-1 derived M0 macrophages treated with TGF-β (10 ng/ml) and CXCL3 (50 ng/ml). All experiments were independently repeated at least three times with consistent results. Data are presented as mean ± SD, and statistical analyses were performed using t test. *p<0.05, **p<0.01.

To further elucidate the interaction mechanisms between IGLC3^+^ tumor cell subpopulations and macrophages, we analyzed the diversity of macrophage populations in CRC tissues. scRNA-seq of tumor and adjacent normal tissues identified 1,350 macrophages, which were clustered into five distinct subtypes: C1QC&LYVE1^+^ macrophages, C1QC^+^ macrophages, C1QC&ISG15^+^ macrophages, ISG15^+^ macrophages, and SPP1^+^ macrophages (**Figure 3A**; **Supplementary Figures 2B, C**), each exhibiting unique gene expression profiles (**Figure 3B**). Analysis of inflammatory marker gene expression revealed that SPP1^+^ macrophages exhibited an anti-inflammatory, M2-like phenotype, while ISG15^+^ macrophages showed pro-inflammatory, M1-like characteristics (**Figure 3C**). Pseudotime analysis indicated that both SPP1^+^ and ISG15^+^ subtypes had distinct differentiation trajectories (**Figure 3D**). To evaluate the role of tumor-derived IGLC3 in macrophage polarization, we co-cultured THP-1-derived M0 macrophages with either vector control or IGLC3-overexpressing HCT116/SW480 cells (**Figure 3E**). After 72 hours, expression of C1QC, LYVE1, SPP1, and ISG15 in macrophages was assessed. Macrophages co-cultured with IGLC3^-^ tumor cells showed significant upregulation of SPP1, whereas those co-cultured with IGLC3-overexpressing cells exhibited modest ISG15 induction but no SPP1 increase (**Figure 3F**). This suggests that IGLC3^-^ tumor cells promote the differentiation of macrophages into an SPP1^+^ phenotype. Previous studies indicate that SPP1^+^ macrophages resemble M2-like macrophages and are associated with poor prognosis in lung carcinoma (13), whereas ISG15^+^ macrophages exhibit M1-like characteristics (22). In our study, SPP1^+^ macrophages, isolated from freshly dissociated tumor tissues using flow cytometry based on CD68 and SPP1 double-positive gating, were compared with M0 and M2 macrophages induced from peripheral blood. SPP1^+^ macrophages derived from patient tumors expressed high levels of M2-associated markers, including CD206, IL-10, and Arg1, though they did not fully overlap with classical M2 macrophages (**Figure 3G**). We hypothesize that IGLC3^-^ tumor cells drive the differentiation of M0 macrophages into SPP1^+^ macrophages, thereby promoting tumor progression. To test this hypothesis, we performed immunofluorescence staining of CD68 and SPP1 in CRC tissues to assess the distribution of SPP1^+^ macrophages. High-stage CRC tissues contained a greater accumulation of SPP1^+^ macrophages, correlating with increased metastasis risk and potential drug resistance (**Figure 3H**). These findings suggest a link between SPP1^+^ macrophages and poor prognosis in CRC patients. Next, we compared cytokine-related gene expression between IGLC3^+^ and IGLC3^-^ tumor cell subpopulations identified from our scRNA-seq dataset (GSE166555). IGLC3^+^ tumor cells exhibited higher expression of CXCL3 and TNFRSF17, whereas IGLC3^-^ tumor cells showed elevated levels of IL18 and TGF-β (**Figure 3I**). Previous research indicates that CXCL3 promotes macrophage polarization toward a pro-inflammatory phenotype, while TGF-β drives polarization toward an anti-inflammatory phenotype (23). Therefore, we hypothesize that IGLC3^-^ tumor cells secrete TGF-β to induce SPP1^+^ macrophage polarization, whereas IGLC3^+^ tumor cells secrete CXCL3 to promote ISG15^+^ macrophage differentiation. To validate this hypothesis, we quantified TGF-β and CXCL3 secretion by vector and IGLC3-overexpressing HCT116/SW480 cells using ELISA. Overexpression of IGLC3 reduced TGF-β secretion (**Figure 3J**) but increased CXCL3 secretion (**Figure 3K**). Treatment of M0 macrophages with recombinant TGF-β significantly upregulated SPP1 expression, whereas CXCL3 induced ISG15 and increased M1 markers CD80 and CD86 (**Figure 3L**). Together, these findings indicate that IGLC3^-^ tumor cells secrete TGF-β to polarize macrophages into an SPP1^+^, M2-like state, contributing to a pro-tumor immune microenvironment.

### 3.4 SPP1^+^ macrophages facilitate CRC stemness

**Figure 4:**
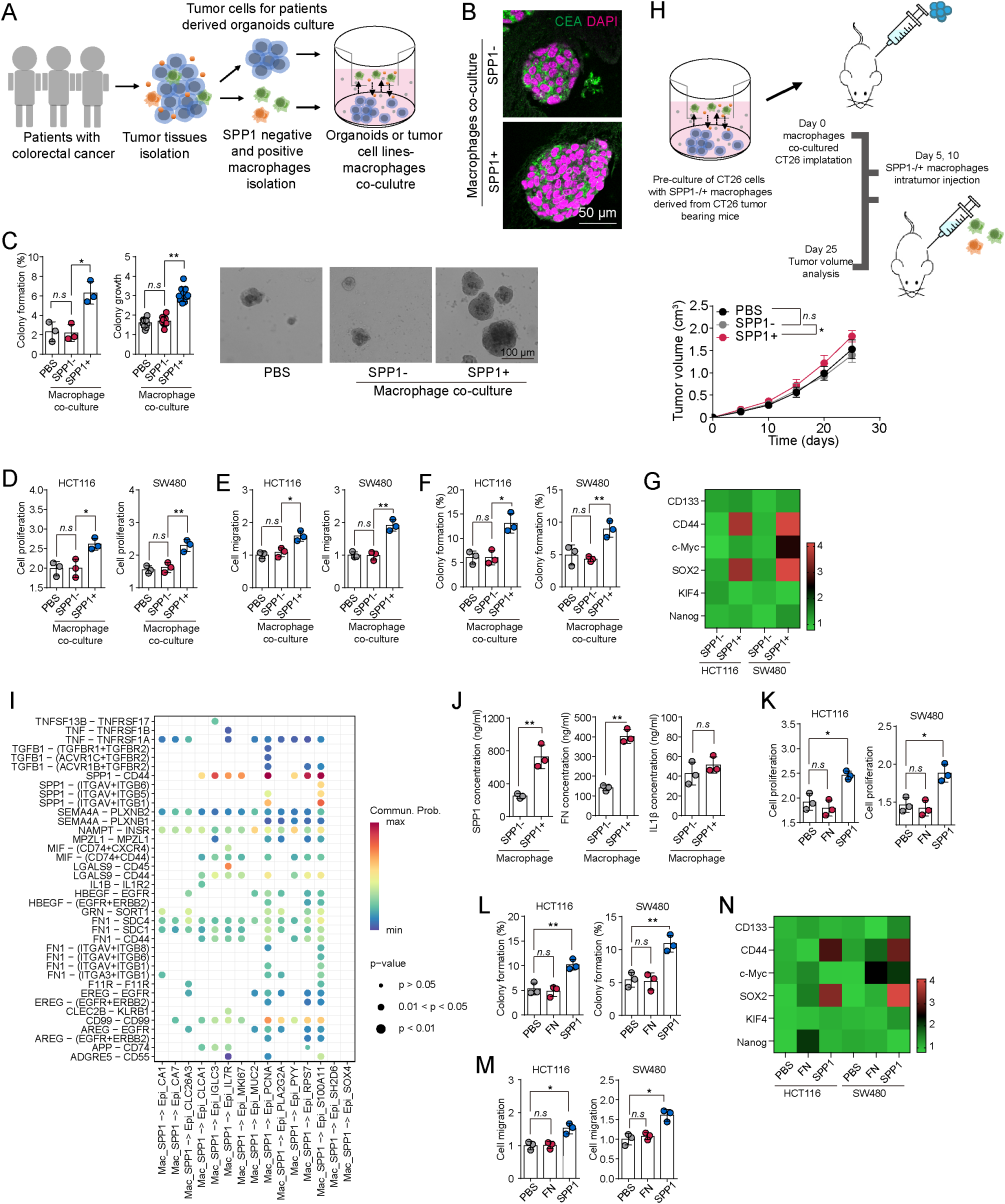
SPP1^+^ macrophages facilitate CRC stemness. **(A)** Schematic diagram of macrophage and tumor cells co-culture model. **(B)** Immunostaining of CEA in patient-derived colorectal tumor tissues co-cultured with SPP1^-^ macrophages or SPP1^+^ macrophages. **(C)** Colony formation capability and growth of patient-derived colorectal tumor tissues co-cultured with SPP1^-^ macrophages or SPP1^+^ macrophages. **(D-G)** Cell proliferation **(D)**, migration **(E)**, colony formation capability **(F)** and stem gene expression (CD133, CD44, SOX2, c-Myc, KlF4, Nanog) **(G)** of HCT116/SW480 cells co-cultured with PBS, SPP1^-^ macrophages or SPP1^+^ macrophages. **(H)** CT26 cells were co-cultured with CT26-bearing mice derived SPP1^-^ macrophages or SPP1^+^ macrophages, and subcutaneously injected into BalB/C mice. On day 5 and 10, mice were treated with SPP1^-^ macrophages or SPP1^+^ macrophages. Tumor volume was determined (n=5 in each group). **(I)** Bubble plots represent important ligand receptor pairs between SPP1^+^ macrophages and 15 tumors subtypes. **(J)** SPP1, FN and IL-1β were quantified in supernatant of SPP1^-^ macrophages or SPP1^+^ macrophages by Elisa. **(K-N)**, Cell proliferation **(K)**, migration **(L)**, colony formation capability **(M)** and stem gene expression (CD133, CD44, SOX2, c-Myc, KlF4, Nanog) **(N)** of HCT116/SW480 cells treated with PBS, SPP1 (500 ng/ml) and FN (500 ng/ml). All experiments were independently repeated at least three times with consistent results. Data are presented as mean ± SD, and statistical analyses were performed using t test. *p<0.05, **p<0.01.

To explore how SPP1^+^ macrophages influence tumor cell behavior, we established a 3D co-culture model using patient-derived colorectal cancer organoids and flow cytometry-isolated SPP1^+^ or SPP1^-^ macrophages from tumor tissues at a 1:10 ratio. CEA staining confirmed the successful cultivation of these tumor organoids (**Figure 4A**). Organoids co-cultured with SPP1^+^ macrophages exhibited markedly accelerated growth, as confirmed by CEA immunostaining (**Figure 4B**). When dissociated and subjected to a secondary 3D colony formation assay, these organoids also showed significantly enhanced clonogenic potential (**Figure 4C**). Consistent with these findings, co-culture with SPP1^+^ macrophages promoted proliferation and migration of HCT116 and SW480 cells (**Figures 4D, E**). Additionally, these cells exhibited increased colony formation and elevated expression of stemness-related genes, including CD44 and SOX2 (**Figures 4F, G**; **Supplementary Figure 2D**). Additionally, to obtain an unbiased and global view of the transcriptional landscape, we performed RNA-seq in CRC cells treated with SPP1^+/-^ macrophages, followed by Gene Set Enrichment Analysis. The results revealed significant enrichment of NOTCH signaling and Wnt/β-catenin signaling, both of which are well-established pathways involved in cancer stemness and self-renewal (**Supplementary Figure 2E**). To validate these results *in vivo*, we further isolated SPP1^+^ and SPP1^-^ macrophages from tumor tissues of CT26 tumor-bearing Balb/c mice and co-cultured them with CT26 cells. The CT26 cells were subsequently transplanted into Balb/c mice to establish a subcutaneous tumor model. Mice treated with SPP1^+^ macrophages on days 5 and 10 after implantation developed significantly larger tumors than those receiving SPP1^-^ macrophages (**Figure 4H**), further confirming the tumor-promoting effect of SPP1^+^ macrophages. These results collectively indicate that SPP1^+^ macrophages are critical in promoting colorectal cancer cell stemness, tumor growth, migration, and colony formation. Next, we investigated the mechanism by which SPP1^+^ macrophages enhance tumor cell stemness.

We conducted scRNA-seq-based ligand–receptor interaction analysis between SPP1^+^ macrophages and the 15 identified tumor cell subtypes. SPP1–CD44 and FN–integrin were among the top-ranked interaction pairs, suggesting their involvement in macrophage–tumor crosstalk (**Figure 4I**). ELISA confirmed that SPP1^+^ macrophages secreted significantly higher levels of SPP1 and fibronectin (FN), but not IL-1β, compared to SPP1^-^ macrophages (**Figure 4J**). We next treated HCT116 and SW480 cells with recombinant SPP1 or FN to determine their individual effects. While FN had negligible influence, SPP1 treatment significantly promoted proliferation, migration, colony formation, and expression of stemness markers such as CD44, SOX2, KLF4, Nanog, and c-Myc (**Figures 4K–N**). These findings indicate that SPP1^+^ macrophages enhance tumor cell stemness and malignant behavior primarily through SPP1 secretion, rather than fibronectin or other cytokines.

### 3.5 BTF3 is the key downstream factor in SPP1-meidated CRC progression

**Figure 5:**
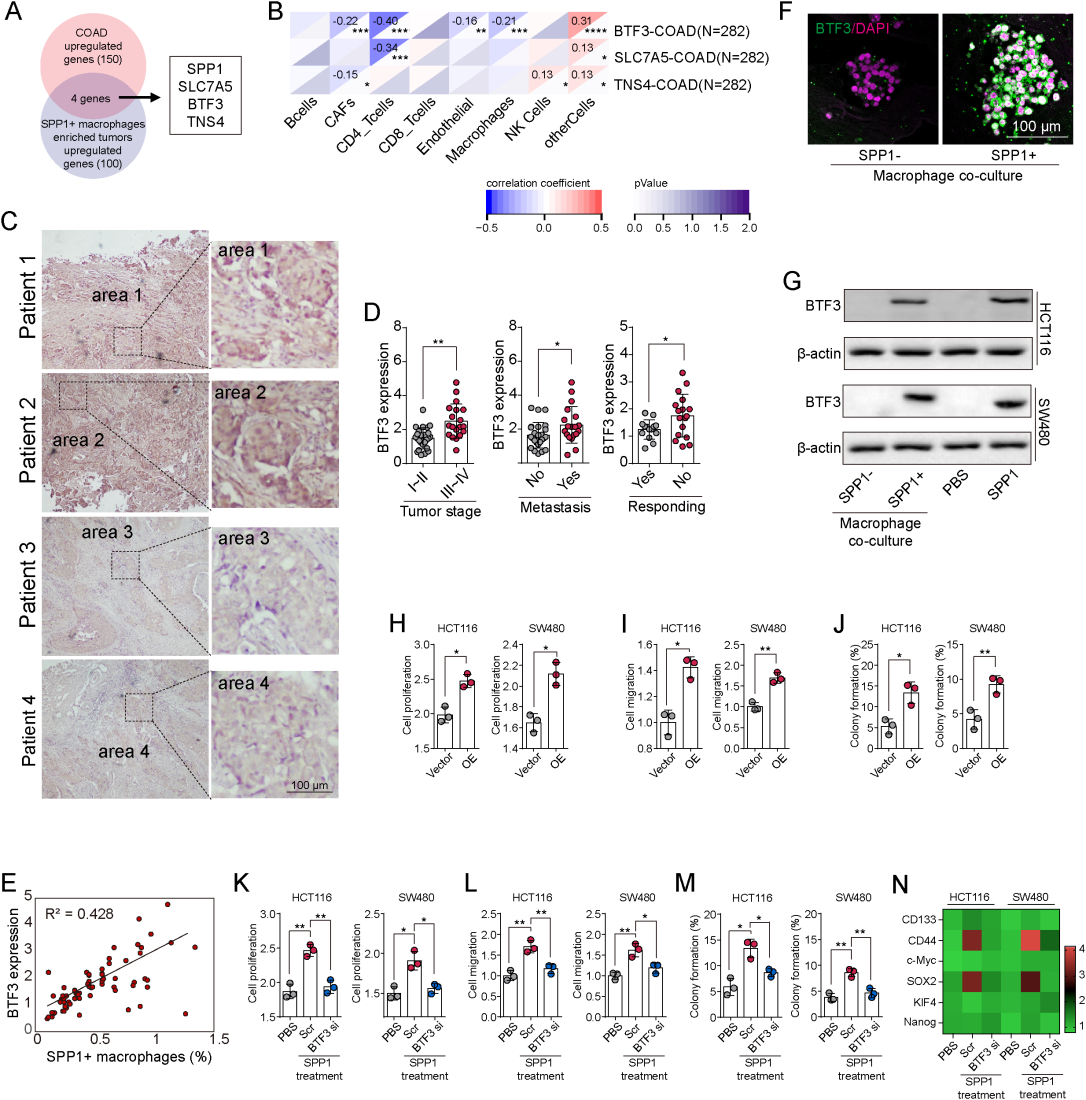
BTF3 is the key downstream factor in SPP1-meidated CRC progression. **(A)** SPP1, SLC7A5, BTF3 and TNS4 were identified as the potential oncogenes in SPP1^+^ macrophages associated CRC progression. **(B)** Correlation analysis between BTF3/SLC7A5/TNS4 expression and each immune cell subtype in CRC patients from TCGA database. **(C)** Immunofluorescence staining of BTF3 in patient-derived colorectal tumor tissues co-cultured with SPP1^-^ macrophages or SPP1^+^ macrophages. **(D)** The BTF3 expression intensity was compared between patients with stage I~II (low stage) or stage III~IV (high stage), with metastasis or not, and responding to chemotherapy or not. **(E)** The correlation analysis between BTF3 expression and SPP1^+^ macrophages distribution in 75 patients with colorectal cancer. **(F)** Immunostaining of BTF3 in patient-derived organoids co-cultured with SPP1^-^ macrophages or SPP1^+^ macrophages. **(G)** Western blotting of BTF3 in HCT116/SW480 cells co-cultured with SPP1^-^ macrophages and SPP1^+^ macrophages, or PBS and SPP1 (500 ng/ml) treated HCT116/SW480 cells. **(H-J)** Cell proliferation **(H)**, migration **(I)** and colony formation capability **(J)** in vector and BTF3 overexpressed HCT116/SW480 cells. K~N, HCT116/SW480 cells were treated with PBS, SPP1 (500 ng/ml), or SPP1 (500 ng/ml) combining BTF3 siRNAs. Cell proliferation **(K)**, migration **(L)**, colony formation capability **(M)** and stem gene expression (CD133, CD44, SOX2, c-Myc, KlF4, Nanog) **(N)** were then determined. All experiments were independently repeated at least three times with consistent results. Data are presented as mean ± SD, and statistical analyses were performed using t test. *p<0.05, **p<0.01, ***p<0.001, ****p<0.0001.

To identify downstream effectors mediating the pro-tumor functions of SPP1^+^ macrophages, we performed a two-step gene filtering strategy. First, we compared transcriptomic profiles between tumors with high and low infiltration of SPP1^+^ macrophages from 13 CRC patients, identifying the top 150 differentially expressed genes. Then, we intersected these genes with the top 100 genes differentially expressed between tumor and normal tissues in the TCGA dataset. Four candidates—SPP1, SLC7A5, BTF3, and TNS4—were identified as potential mediators (**Figure 5A**). Among these, only BTF3 showed a strong positive correlation with macrophage infiltration in TCGA-COAD data (**Figure 5B**). As a transcription factor implicated in stemness and cell proliferation (24), BTF3 was prioritized for further analysis. Immunohistochemical staining of clinical CRC samples revealed elevated BTF3 expression in high-stage tumors, metastatic cases, and non-responders to chemotherapy (**Figures 5C, D**). Moreover, BTF3 expression positively correlated with SPP1^+^ macrophage infiltration across patient samples (**Figure 5E**), suggesting a functional link. To test whether SPP1^+^ macrophages regulate BTF3 expression, we analyzed BTF3 levels in patient-derived CRC organoids and CRC cell lines (HCT116, SW480) following co-culture with SPP1^+^ or SPP1^-^ macrophages. BTF3 expression was significantly upregulated by SPP1^+^ macrophages, as shown by PCR and western blotting (**Figures 5F, G**; **Supplementary Figure 3A**). Similarly, direct treatment with recombinant SPP1 increased BTF3 protein levels in tumor cells. Functionally, BTF3 overexpression enhanced proliferation, migration, and colony formation in HCT116 and SW480 cells (**Figures 5H–J**). Conversely, silencing BTF3 with siRNAs (**Supplementary Figure 3B**) abolished SPP1-induced tumor cell growth, migration, colony formation, and expression of key stemness markers, including CD133, CD44, SOX2, c-Myc, Nanog, and KLF4 (**Figures 5K–N**). These results establish BTF3 as a critical downstream effector of SPP1^+^ macrophage-induced tumor progression and stemness in colorectal cancer.

### 3.6 SPP1^+^ macrophages mediate BTF3 upregulated through CD44/Wnt signaling pathway

**Figure 6:**
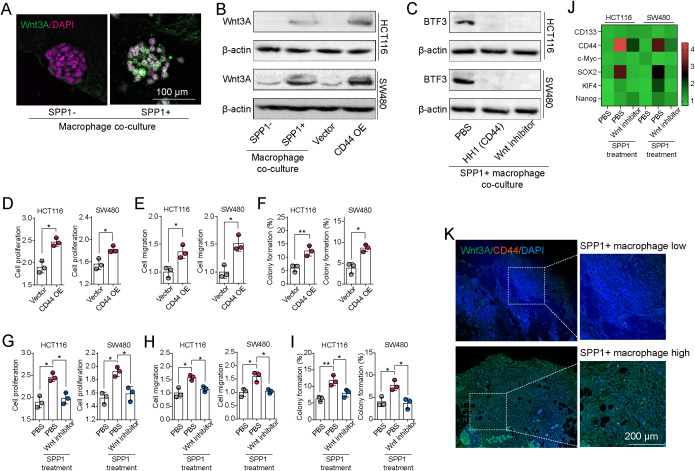
SPP1^+^ macrophages mediate BTF3 upregulated through CD44/Wnt signaling pathway. **(A)** Immunostaining of Wnt3A in patient-derived organoids co-cultured with SPP1^-^ macrophages or SPP1^+^ macrophages. **(B)** Western blotting of Wnt3A in HCT116/SW480 cells co-cultured with SPP1^-^ macrophages and SPP1^+^ macrophages, or vector and CD44 overexpressed HCT116/SW480 cells. **(C)** HCT116/SW480 cells were co-cultured with SPP1^+^ macrophages, then treated with PBS, HH1 (50 nM) or Wnt inhibitor (20 nM). The expression of BTF3 in HCT116/SW480 cells was then determined by western blotting. **(D-F)** Cell proliferation **(D)**, migration **(E)** and colony formation capability **(F)** in vector and CD44 overexpressed HCT116/SW480 cells. **(G-J)** HCT116/SW480 cells were treated with PBS, SPP1 (500 ng/ml), or SPP1 (500 ng/ml) combining Wnt inhibitor (20 nM). Cell proliferation **(G)**, migration **(H)**, colony formation capability **(I)** and stem gene expression (CD133, CD44, SOX2, c-Myc, KlF4, Nanog) **(J)** were then determined. **(K)** Immunostaining of CD44 and Wnt3A in tumor tissues from SPP1^+^ macrophage high and low CRC patients. All experiments were independently repeated at least three times with consistent results. Data are presented as mean ± SD, and statistical analyses were performed using t test. *p<0.05, **p<0.01.

We next investigated how SPP1^+^ macrophages induce BTF3 expression in tumor cells. Prior studies and our ligand–receptor analysis suggested that SPP1 can bind to CD44 on tumor cells, triggering downstream oncogenic signaling. CD44 is known to regulate stemness and gene transcription via activation of the Wnt3A pathway (25). We first examined Wnt3A expression in patient-derived CRC organoids co-cultured with SPP1^+^ or SPP1^-^ macrophages. Wnt3A levels were significantly elevated in organoids exposed to SPP1^+^ macrophages (**Figure 6A**). Similarly, in HCT116 and SW480 cells, both co-culture with SPP1^+^ macrophages and CD44 overexpression upregulated Wnt3A expression (**Figure 6B**). To clarify whether CD44/Wnt signaling mediates BTF3 upregulation, we treated SPP1^+^ macrophage–co-cultured CRC cells with either the CD44 inhibitor HH1 or a Wnt inhibitor. Both treatments effectively suppressed BTF3 expression (**Figure 6C** and **S3C**), confirming the involvement of this signaling cascade. Functionally, CD44 overexpression (**Supplementary Figure 3D**) promoted proliferation (**Figure 6D**), migration (**Figure 6E**), and colony formation (**Figure 6F**). Moreover, stimulation with recombinant SPP1 enhanced all these tumorigenic behaviors and upregulated multiple stemness markers, including CD133, CD44, SOX2, c-Myc, KLF4, and Nanog. Notably, these effects were largely reversed by Wnt inhibition or CD44 siRNAs (**Figures 6G–J**; **Supplementary Figures 3E-H**). Finally, immunohistochemical analysis of CRC patient samples revealed that tumors with high SPP1^+^ macrophage infiltration also exhibited elevated expression of both CD44 and Wnt3A (**Figure 6K**), further supporting the *in vivo* relevance of the SPP1–CD44–Wnt–BTF3 axis. Together, these data demonstrate that SPP1^+^ macrophages enhance tumor progression and stemness through CD44-mediated activation of Wnt signaling, which in turn upregulates BTF3 expression in CRC cells.

### 3.7 Inhibit IGLC/SPP1/CD44/BTF3/Wnt signaling to overcome chemoresistance in colorectal cancer

**Figure 7:**
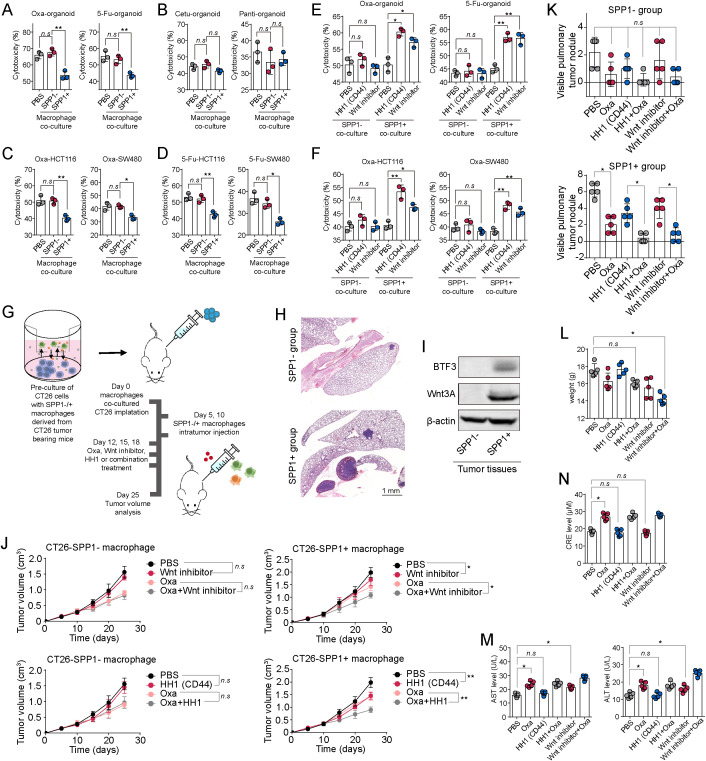
Inhibit IGLC/SPP1/CD44/BTF3/Wnt signaling to overcome chemoresistance in colorectal cancer. **(A)** Cytotoxicity of Oxa (5 μM, 48 hours) or 5-Fu (10 μM, 48 hours) to patients derived organoids co-cultured with PBS, SPP1^-^ macrophages or SPP1^+^ macrophages. **(B)** Cytotoxicity of Cetuximab (20 nM, 48 hours) or Panitumumab (20 nM, 48 hours) to patients derived organoids co-cultured with PBS, SPP1^-^ macrophages or SPP1^+^ macrophages. **(C, D)**, Cytotoxicity of Oxa (5 μM, 48 hours) or 5-Fu (10 μM, 48 hours) to HCT116/SW480 cells co-cultured with PBS, SPP1^-^ macrophages or SPP1^+^ macrophages. **(E)** Patients derived organoids were co-cultured with SPP1^-^ macrophages or SPP1^+^ macrophages. Then organoids were treated with Oxa (5 μM, 48 hours) combining PBS, HH1 (50 nM), Wnt inhibitor (20 nM) or 5-Fu (10 μM, 48 hours) combining PBS, HH1 (50 nM), Wnt inhibitor (20 nM). Cell apoptosis was determined. **(F)** HCT116 or SW480 cells were co-cultured with SPP1^-^ macrophages or SPP1^+^ macrophages. Then HCT116 or SW480 cells were treated with Oxa (5 μM, 48 hours) combining PBS, HH1 (50 nM), Wnt inhibitor (20 nM). Cell apoptosis was determined. **(G)** CT26 cells were co-cultured with SPP1^-^ macrophages or SPP1^+^ macrophages. Then CT26 cells were subcutaneously injected to BALB/c mice. On day 5 and 10, mice were treated with SPP1^-^ macrophages or SPP1^+^ macrophages. On day 12, 15 and 18, mice were treated with Oxa, Wnt inhibitor, HH1 or combination (n=5 in each group). **(H)** H&E staining of lung tissues from mice (G, day 20). **(I)** Western blotting of BTF3 and Wnt3A in tumor tissues from mice (G, day 20). **(J)** Tumor volume of tumor-baring mice **(G)**. **(K)** Pulmonary tumor nodules count in mice **(G)**. **(L, M)** Healthy BALB/c mice were treated with Oxa, HH1, Wnt inhibitor or combination. The body weight **(L)**, serum CRE **(N)**, AST and ALT **(M)** level of mice were determined (n=5 in each group). All experiments were independently repeated at least three times with consistent results. Data are presented as mean ± SD, and statistical analyses were performed using t test. *p<0.05, **p<0.01.

Our earlier findings showed that SPP1^+^ macrophages are enriched in chemoresistant CRC tissues. This finding led us to hypothesize that the IGLC3^-^ tumor cell–SPP1^+^ macrophage axis contributes to chemoresistance in CRC, particularly by modulating CD44–Wnt–BTF3 signaling. To test this, we co-cultured patient-derived CRC organoids with either SPP1^+^ or SPP1^-^ macrophages and treated them with standard chemotherapeutic agents 5-Fu and oxaliplatin. Organoids exposed to SPP1^+^ macrophages exhibited significant resistance to both drugs (**Figure 7A**). However, sensitivity to targeted agents cetuximab and panitumumab, which act via the EGFR pathway, was not affected (**Figure 7B**), suggesting pathway specificity. Similar chemoresistance patterns were observed in HCT116 and SW480 cells co-cultured with SPP1^+^ macrophages (**Figures 7C, D**). To evaluate whether blocking the CD44–Wnt axis could reverse this resistance, we treated co-cultures with the CD44 inhibitor HH1 or a Wnt inhibitor in combination with chemotherapy. Neither HH1 nor the Wnt inhibitor significantly affected SPP1^-^ macrophage co-cultures. However, in the presence of SPP1^+^ macrophages, both agents effectively sensitized tumor cells to 5-Fu and Oxa, as indicated by increased apoptosis (**Figures 7E, F**).

*In vivo*, we established CRC models in BALB/c mice using CT26 cells preconditioned by co-culture with SPP1^+^ or SPP1^-^ macrophages. Mice bearing SPP1^+^-primed tumors exhibited faster tumor growth, elevated lung metastasis, and higher levels of BTF3 and Wnt3A (**Figures 7G–I**). Treatment with Oxa alone had limited efficacy in the SPP1^+^ group. However, combination therapy with HH1 or the Wnt inhibitor significantly enhanced tumor suppression and reduced pulmonary metastasis (**Figures 7J, K**). To assess safety, we administered HH1 or the Wnt inhibitor in combination with Oxa to healthy BALB/c mice. While both agents were well tolerated in terms of liver enzymes, mice receiving the Wnt inhibitor showed significant weight loss and elevated AST/ALT levels, indicating potential systemic toxicity. In contrast, HH1 had a more favorable safety profile (**Figures 7L–N**). Collectively, these results demonstrate that the IGLC3^-^/SPP1^+^ macrophage axis promotes chemoresistance in CRC via CD44–Wnt–BTF3 signaling, and that targeting this pathway—particularly with HH1—offers a promising therapeutic approach to overcome resistance while minimizing systemic toxicity.

## 4 Discussion

In this study, we uncover a previously unrecognized mechanism by which IGLC3^-^ tumor cells reshape the tumor microenvironment to promote CRC progression and chemoresistance. Specifically, IGLC3^-^ tumor cells secrete TGF-β to polarize macrophages into an SPP1^+^, M2-like state, which in turn promotes tumor cell proliferation, stemness, and therapy resistance through activation of the CD44–Wnt–BTF3 signaling axis. By integrating scRNA-seq data, patient-derived organoids, and *in vivo* models, our work reveals a critical tumor–macrophage interaction network that drives malignancy and therapeutic failure in CRC.

CRC is characterized by extensive intratumoral heterogeneity, which underlies variable responses to treatment (26). Through single-cell transcriptomics, we identified two epithelial subpopulations—CLCA1^+^ and IGLC3^+^ tumor cells—that were both associated with patient prognosis. Among them, CLCA1^+^ cells were more abundant and showed a stronger statistical correlation with favorable survival outcomes. However, the role of CLCA1 in colorectal cancer has been extensively reported, particularly in regulating epithelial differentiation and tumor cell proliferation. In contrast, the function of IGLC3 in cancer biology remains entirely unexplored. Given this knowledge gap, we focused our downstream analysis on IGLC3-defined tumor subsets to explore novel biological mechanisms. We identified IGLC3^+^ and IGLC3^-^ tumor subpopulations with distinct prognostic and functional properties. IGLC3^+^ cells were associated with favorable clinical outcomes, whereas IGLC3^-^ cells correlated with advanced disease, poor treatment response, and increased interaction with macrophages. Although IGLC3^+^ epithelial cells account for a relatively small proportion of the total tumor population, our study primarily focuses on the IGLC3^-^ tumor cells, which represent the dominant subpopulation and actively contribute to colorectal cancer progression. These IGLC3^-^ cells promote tumor-supportive macrophage polarization and activate pro-tumor signaling pathways such as the CD44–Wnt–BTF3 axis. Notably, the rarity of IGLC3^+^ cells does not preclude their biological relevance, as their absence marks a tumor subtype associated with more aggressive behavior and worse prognosis. This highlights the functional importance of IGLC3-based tumor classification, despite the low abundance of IGLC3^+^ cells. This underscores the importance of IGLC3-based tumor classification within CRC tumors, not only for prognostic stratification but also for mechanistic understanding.

Macrophages are highly plastic immune cells whose polarization states critically influence the tumor microenvironment (27, 28). Our findings demonstrate that IGLC3^-^ tumor cells polarize macrophages into an SPP1^+^ phenotype, a distinct TAM subset that shares features with M2-like macrophages and is strongly associated with immunosuppression, tumor growth, and drug resistance (29). This process is driven by tumor-derived TGF-β, while IGLC3^+^ tumor cells secrete CXCL3 to promote an ISG15^+^, M1-like macrophage phenotype, indicating a subtype-specific modulation of immune contexture. Intriguingly, SPP1-expressing macrophages have recently emerged as a pro-tumorigenic subset across solid malignancies. Clinically, infiltration of SPP1^+^ macrophages correlates with poor patient outcomes, enhanced invasiveness, and treatment resistance across diverse cancers, including lung adenocarcinoma and colorectal cancer (13, 30). Recent single-cell transcriptomic studies also identified SPP1^+^ TAMs as a conserved macrophage population enriched in advanced tumors, frequently associated with extracellular matrix remodeling and immune suppression (31, 32). SPP1^+^ macrophages in our study were shown to exhibit M2-like characteristics, including elevated expression of CD206, IL-10, and Arg1 (**Figure 3G**), and were significantly enriched in advanced-stage, metastatic, and chemoresistant tumors (**Figure 3H**). Notably, these macrophages were preferentially induced by IGLC3^-^ tumor cells via the secretion of TGF-β (**Figures 3I, J**), a well-established immunoregulatory cytokine known to suppress cytotoxic T lymphocyte (CTL) responses and enhance regulatory T cell (Treg) function. Given these findings, it is plausible that SPP1^+^ TAMs not only facilitate tumor cell plasticity but also establish an immunosuppressive milieu by inhibiting CD8^+^ T cell infiltration, survival, and effector activity. Prior studies have demonstrated that SPP1, in conjunction with M2-related cytokines such as TGF-β and IL-10, can impair antigen presentation, promote T cell exhaustion, and upregulate inhibitory immune checkpoints including PD-L1 and Galectin-9 on macrophages and other myeloid cells. While our current study focused on the macrophage–tumor cell axis, we recognize that the impact of SPP1^+^ macrophages on adaptive immune responses, particularly CD8^+^ T cells, represents a critical and complementary mechanism of tumor immune evasion. To fully elucidate these effects, future investigations should incorporate multiplexed immune profiling, spatial transcriptomic analyses, and functional assays evaluating CD8^+^ T cell activity in the context of SPP1^+^ macrophage enrichment or depletion. *In vivo* studies employing CD8^+^ T cell blockade or depletion, in combination with macrophage-targeted therapies (e.g., anti-SPP1 or anti-TGF-β), may also provide mechanistic insights and therapeutic guidance. Collectively, these perspectives highlight that SPP1^+^ TAMs function as central orchestrators not only of tumor cell stemness but also of immune suppression, and underscore the potential of targeting this macrophage subset to restore anti-tumor immunity and enhance treatment efficacy.

We further reveal that SPP1^+^ macrophages enhance tumor cell stemness via the CD44–Wnt–BTF3 pathway. Lgr5 is a canonical Wnt-associated stem cell marker in colorectal cancer. However, tumor stemness is increasingly recognized as a dynamic and microenvironment-driven state rather than being confined to a single CSC population. In our study, CD44, but not Lgr5, emerged as a dominant receptor mediating interactions between SPP1^+^ macrophages and malignant tumor cells, showing broader expression and a closer association with macrophage-derived SPP1 signaling and Wnt activation. Nevertheless, potential regulation of Lgr5^+^ tumor cells by SPP1^+^ macrophages warrants further investigation. SPP1 binding to CD44 triggers Wnt3A upregulation and activates downstream transcriptional programs, notably BTF3, a transcription factor implicated in stemness and chemoresistance (24). This signaling cascade promotes tumor cell plasticity, growth, and metastasis, and represents a key axis of macrophage-induced malignancy in CRC. Although our results support a functional link between SPP1–CD44–Wnt signaling and BTF3 upregulation in colorectal cancer, the precise molecular mechanism remains to be fully elucidated. The observation that CD44 functions upstream of Wnt signaling in our study merits further discussion, as CD44 is canonically recognized as a downstream target of the Wnt/β-catenin pathway (33). However, accumulating evidence suggests a more complex, bidirectional relationship. Several studies have reported that CD44 can activate Wnt signaling through various mechanisms, such as interacting with and stabilizing the Wnt coreceptor LRP6 (34). These findings support the existence of a forward feedback loop wherein ligand-stimulated CD44 reinforces Wnt signaling. In our study, we demonstrate that macrophage-derived SPP1 binds to CD44 on CRC cells, leading to Wnt3A upregulation and subsequent BTF3-mediated stemness and chemoresistance. This aligns with the emerging concept of CD44 as a context-dependent upstream modulator of Wnt signaling in response to microenvironmental cues. Thus, rather than contradicting established literature, our findings reveal a novel SPP1–CD44–Wnt axis operating within the tumor-immune ecosystem, highlighting the bidirectional complexity of this signaling network. BTF3 does not appear to be a direct transcriptional target of the β-catenin/TCF complex, as suggested by the absence of BTF3 in published TCF/LEF gene sets. This indicates that Wnt signaling may regulate BTF3 expression through indirect pathways. One possibility involves c-Myc, a canonical Wnt target that has been reported to interact with or regulate BTF3 in other tumor contexts. However, in our study, c-Myc levels did not significantly change upon SPP1 or Wnt activation, suggesting that a Wnt–c-Myc–BTF3 axis is unlikely to explain the observed upregulation in CRC. An alternative explanation may lie at the epigenetic level. It is plausible that the BTF3 promoter region is subject to epigenetic regulation downstream of Wnt activation, enabling its transcription in a TCF-independent manner. The mechanistic link between Wnt signaling and BTF3 transcription remains to be clarified, and this question will be prioritized in our future research.

Importantly, we demonstrate that targeting CD44 or Wnt signaling reverses SPP1-induced chemoresistance both *in vitro* and *in vivo*. The CD44 inhibitor HH1, in particular, showed significant therapeutic efficacy when combined with oxaliplatin or 5-Fu and exhibited a favorable toxicity profile compared to Wnt inhibitors. These findings suggest that HH1 may be a clinically viable strategy to overcome TAM-mediated drug resistance in CRC.

Despite the strength of our multi-platform approach, several limitations warrant consideration. First, although we identified the SPP1–CD44–Wnt–BTF3 axis as a dominant mechanism, SPP1^+^ macrophages may exert additional effects on angiogenesis, immune evasion, or extracellular matrix remodeling that remain unexplored. Second, the phenotypic plasticity of TAMs in response to therapy or environmental cues warrants further longitudinal studies. Finally, whether this mechanism applies to other tumor types or immune contexts remains an open question deserving further investigation.

In conclusion, this study reveals that IGLC3^-^ tumor cells orchestrate macrophage polarization to establish an immunosuppressive, stemness-promoting microenvironment through the SPP1–CD44–Wnt–BTF3 axis. Therapeutically disrupting this crosstalk—particularly via CD44 inhibition—may offer a promising approach to enhance chemotherapy sensitivity and improve outcomes for CRC patients.

## Data Availability

The original contributions presented in the study are included in the article/**Supplementary Material**. Further inquiries can be directed to the corresponding authors.
