# Supplementary material for: IGLC3- tumor cells drive chemoresistance in colorectal cancer by polarizing SPP1+ macrophages via the CD44-Wnt-BTF3 axis
Source: Front Immunol. 2026 Apr 1;17:1731216. doi: 10.3389/fimmu.2026.1731216 (PMC13079274; doi:10.3389/fimmu.2026.1731216)
Supplement: Supplementary file 1 [file DataSheet1.docx]

**Supplementary information**

**Supplementary figure 1**

**
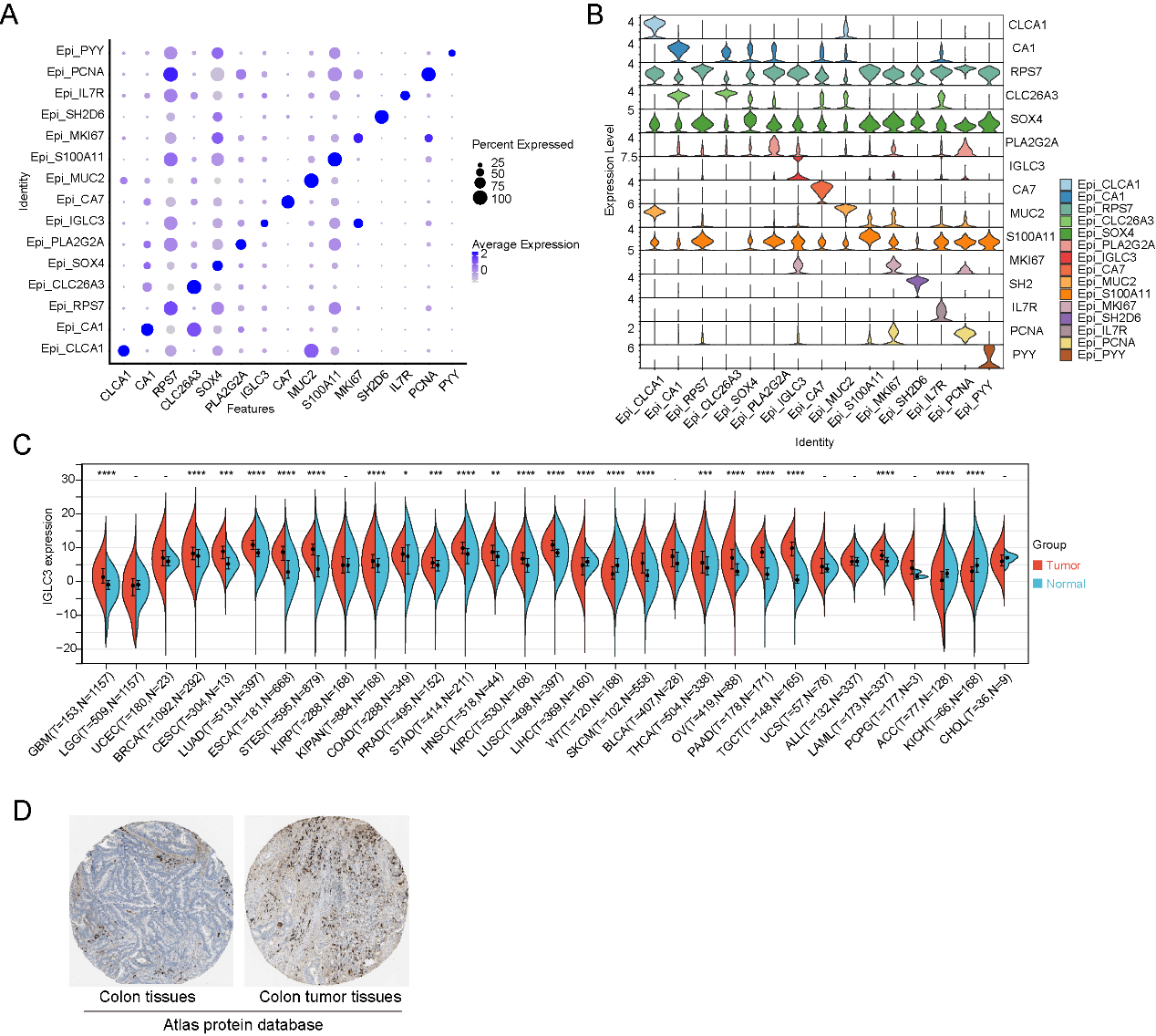
**

Supplementary figure 1

A, The dot plot of 15 tumor cell clusters in scRNA-seq of colorectal tumor tissues, including CLCA1 positive, CLC26A3 positive, PLA2G2A positive, CA1 positive, SH2D6 positive, RPS7 positive, SOX4 positive, CA7 positive, MKI167 positive, MUC2 positive, IGLC3 positive, IL7R positive, PCNA positive, PYY positive, and S100A11 positive cell subtypes. B, the violin plot of 15 tumor cell clusters in scRNA-seq of colorectal tumor tissues, including CLCA1 positive, CLC26A3 positive, PLA2G2A positive, CA1 positive, SH2D6 positive, RPS7 positive, SOX4 positive, CA7 positive, MKI167 positive, MUC2 positive, IGLC3 positive, IL7R positive, PCNA positive, PYY positive, and S100A11 positive cell subtypes. C. mRNA expression of IGLC3 in normal and tumor tissues of patients from TCGA database. D, Immunostaining of IGLC3 in colon tissues and colon tumor tissues of patients from Atlas protein database.

**Supplementary figure 2**


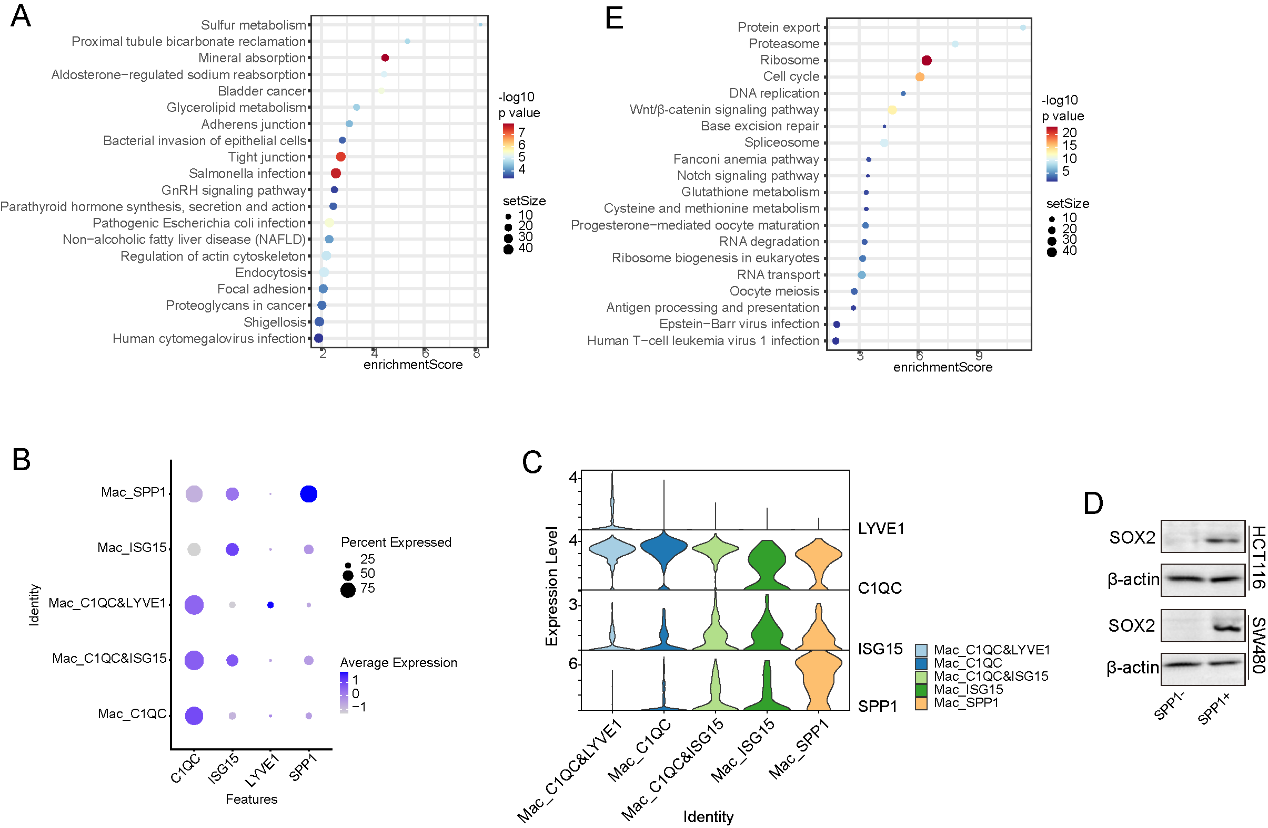


Supplementary figure 2

A. KEGG pathway enrichment analysis of differentially expressed genes between IGLC-positive and IGLC-negative tumor epithelial cell subsets. B, The dot plot of 5 macrophage cell clusters in scRNA-seq of colorectal tumor tissues, including C1QC&LYVE1 positive macrophages, C1QC positive macrophages, C1QC&ISG15 positive macrophages, ISG15 positive macrophages and SPP1 positive macrophages. C, the violin plot of 5 macrophage cell clusters in scRNA-seq of colorectal tumor tissues, including C1QC&LYVE1 positive macrophages, C1QC positive macrophages, C1QC&ISG15 positive macrophages, ISG15 positive macrophages and SPP1 positive macrophages. D, Western blotting of SOX2 in HCT116/SW480 cells co-cultured with SPP1^-/+^ macrophages. E, KEGG pathway enrichment analysis of differentially expressed genes between HCT116 cells co-cultured with SPP1^-/+^ macrophages.

**Supplementary figure 3**


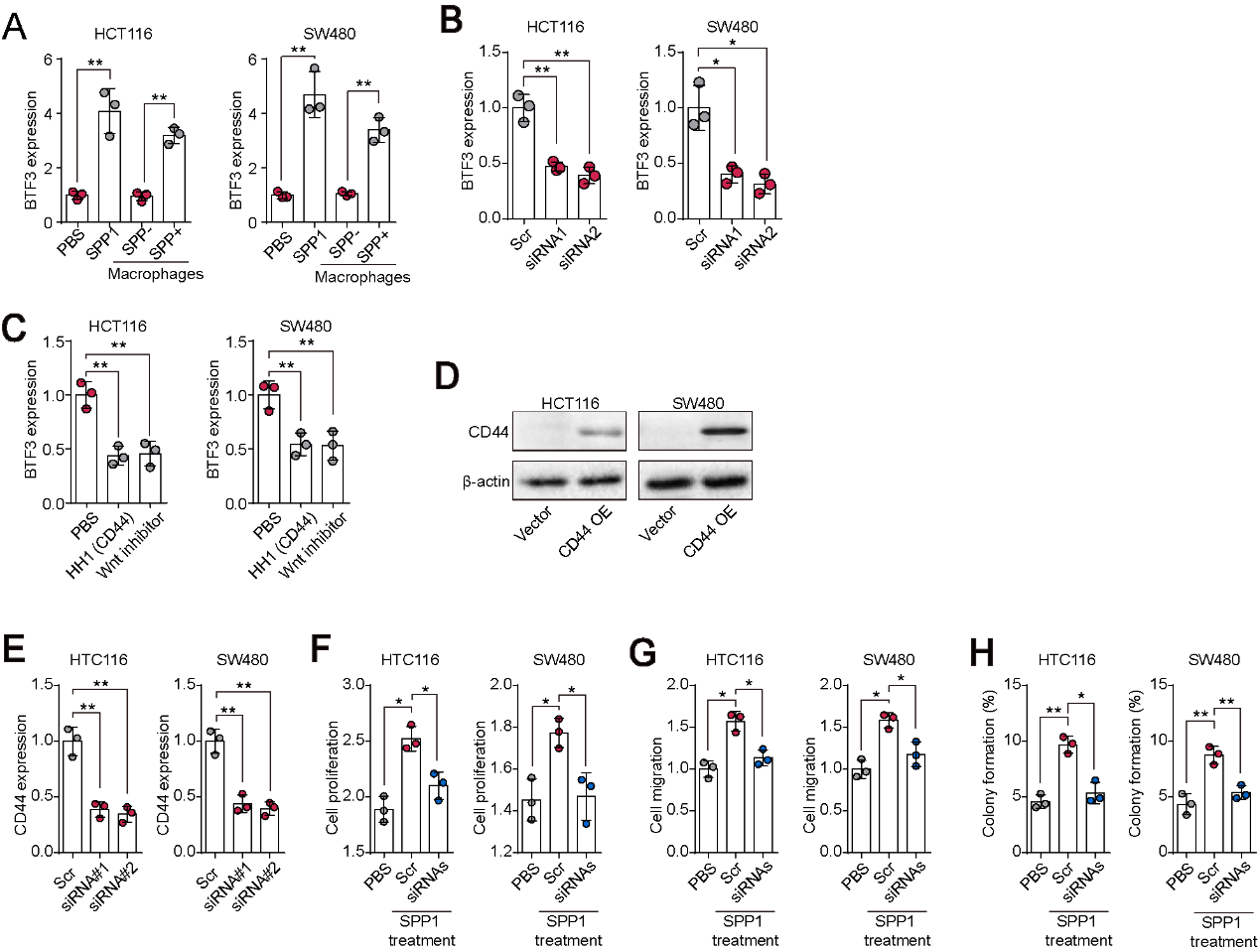


Supplementary figure 3

A, mRNA expression of BTF3 in HCT116/SW480 cells co-cultured with SPP1 negative macrophages and SPP1 positive macrophages, or PBS and SPP1 (500 ng/ml) treated HCT116/SW480 cells. B. mRNA expression of BTF3 in HCT116/SW480 cells treated with scramble or BTF3 siRNAs. C, HCT116/SW480 cells were co-cultured with SPP1 positive macrophages, then treated with PBS, HH1 (50 nM) or Wnt inhibitor (20 nM). The expression of BTF3 at mRNA level in HCT116/SW480 cells was then determined. D, Western blotting of CD44 in vector and CD44 overexpressed HCT116/SW480 cells. E, mRNA expression of BTF3 in HCT116/SW480 cells treated with scramble or CD44 siRNAs. F-H, Cell proliferation (F), migration (G) and colony formation capability (H) of HCT116/SW480 cells treated with PBS, SPP1 (500 ng/ml), or SPP1 (500 ng/ml) combining CD44 siRNAs.

**Supplementary materials and methods:**

1. Single-cell RNA sequencing analysis

Dimensionality Reduction and Clustering: Single-cell data were processed using Seurat (v4.3.0) for normalization, feature selection, dimensionality reduction, and clustering. Gene expression was normalized via the LogNormalize method, which scales each cell's expression relative to the total gene expression within the cell and applies a log-transformation. High-variance genes, exhibiting significant expression variation across cells, were identified using the vst metric, with the top 2000 genes selected for subsequent analysis. Prior to clustering, gene expression values were z-scored to standardize them, ensuring each gene had a mean of 0 and a variance of 1, thus equalizing their contributions and preventing high-expressed genes from dominating. Principal Component Analysis (PCA) was performed, and the top 25 principal components were chosen for clustering. A K-nearest neighbor (KNN) graph was constructed in PCA space, refining cell connections based on Jaccard similarity. Cells were grouped using the Louvain modularity optimization algorithm with a resolution set to 0.4. Nonlinear dimensionality reduction techniques, such as tSNE and UMAP, were employed to visualize the clustering of cells in lower-dimensional space. To address batch effects across multiple datasets, the Harmony algorithm (v0.1.0) was applied. Harmony integrates data by performing K-means clustering to maximize cluster diversity and adjusts principal component coordinates to align with cluster centroids, iterating until convergence.

Cell Type Annotation: Cell types were manually annotated based on classical marker genes reported in the literature.

Cluster-Specific Differential Gene Analysis: Differential gene expression was performed to identify cluster-specific marker genes. Each cell cluster was compared to the remaining cells (one vs. others) to identify upregulated (positive) and downregulated (negative) marker genes. The Wilcoxon Rank Sum Test was used for differential expression analysis. Genes were included if they had a minimum average log2 fold change of 0.25 between two groups and were expressed in at least 10% of cells in either group. Only clusters with at least 3 cells were considered in the analysis.

Trajectory Analysis: Developmental signals between subpopulations were inferred using the R package Monocle2. The top 2000 high-variance genes from the subpopulations were used to order the cells, followed by dimensionality reduction and trajectory fitting using a reverse graph embedding algorithm.

Cell-Cell Communication Analysis: Cell-to-cell communication, mediated by cytokines, membrane proteins, and other factors, forms a complex regulatory network that coordinates biological processes such as development, differentiation, and disease. To assess these interactions, the CellChat (v1.6.1) package was used to evaluate receptor-ligand pairs between cell clusters. Receptor-ligand interactions with a p-value < 0.05 were considered significant.

Survival Analysis: The TCGA-READ (Primary Tumor: 166; Recurrent Tumor: 1; Solid Tissue Normal: 10) and TCGA-COAD (Metastatic: 1; Primary Tumor: 456; Recurrent Tumor: 1; Solid Tissue Normal: 41) datasets were downloaded from the TCGA database (https://portal.gdc.cancer.gov/). Differential genes from the top 50 epithelial cell subgroups were used to calculate GSVA scores in TCGA tumor samples using the GSVA package (v1.52.3). Survival analysis was conducted using the survival package (v3.3.1).

Epithelial Stemness Scoring: Epithelial stemness gene sets were sourced from (http://biocc.hrbmu.edu.cn/CancerSEA/goDownload), and stemness scores were calculated using the GSVA package (v1.52.3) and visualized with the ComplexHeatmap package (v2.14.0).

2. Patient-Derived Organoid Establishment
Fresh colorectal cancer specimens were collected immediately after surgical resection, minced into 1–2 mm³ fragments using sterile scalpels, and enzymatically dissociated in a digestion solution containing 1 mg/mL collagenase IV (Gibco, USA) and 0.1 mg/mL DNase I (Roche, Switzerland) in advanced DMEM/F12 medium (Gibco, USA) for 60 minutes at 37°C with gentle agitation. The cell suspension was filtered through a 22-µm cell strainer (Corning, USA) to remove undigested debris. Cells were pelleted by centrifugation at 300 × g for 5 minutes, washed with PBS, and resuspended in ice-cold Matrigel (Corning, USA). Approximately 30 µL of the Matrigel-cell mixture was plated as a dome in pre-warmed 24-well plates. Once solidified, organoid culture medium containing advanced DMEM/F12, 1× B27 supplement (Gibco, USA), 1.25 mM N-acetylcysteine (Sigma-Aldrich, USA), 50 ng/mL recombinant human epidermal growth factor (PeproTech, USA), 100 ng/mL Noggin (PeproTech, USA), and 500 ng/mL R-spondin-1 (PeproTech, USA) was added. Cultures were maintained at 37°C in a humidified 5% CO₂ incubator, and the medium was replaced every 2–3 days. Organoids were passaged mechanically or enzymatically using TrypLE Express (Gibco, USA) approximately every 7–10 days. The details of patients were shown in table II.

3. Cell Transfection

For overexpression of CD44 and BTF3, recombinant plasmids encoding human CD44 (CD44-OE) and BTF3 (BTF3-OE) were constructed using the pCDNA3.1(+) vector (Invitrogen, Thermo Fisher, USA). For siRNA-mediated knockdown of BTF3, specific siRNAs targeting BTF3 (BTF3 siRNA) were purchased from GenePharma (Shanghai, China). Negative control siRNA (scramble siRNA) was used as a reference. Transfections were carried out using Lipofectamine™ 3000 Transfection Reagent (Invitrogen, Thermo Fisher, USA) according to the manufacturer's instructions. Briefly, 2 μg of plasmid DNA or 50 nM siRNA was mixed with Lipofectamine™ 3000 reagent in Opti-MEM medium (Gibco, Thermo Fisher, USA) and incubated for 5 hours. After transfection, the medium was replaced with fresh RPMI-1640 supplemented with 10% FBS, and cells were incubated for an additional 48 hours before use in downstream assays.

4. Cell Proliferation Assay

Cell proliferation was assessed using the Cell Counting Kit-8 (CCK-8; Dojindo, Japan). HCT116 and SW480 cells (2 × 10³ cells/well) were seeded into 96-well plates. After various treatment durations, 10 μL of CCK-8 solution was added to each well, and the plates were incubated at 37°C for 1 hour. Absorbance at 450 nm was measured using a microplate reader (BioTek, USA).

5. Cell Migration Assay

The migratory capacity of tumor cells was evaluated using 24-well Transwell chambers (8-μm pore size; Corning). Cells were suspended in serum-free medium, and 200 μL of cell suspension (1 × 10⁵ cells) was added to the upper chamber, while the lower chamber contained 600 μL of medium supplemented with 10% FBS. After 24 hours of incubation at 37°C, non-migrated cells were removed from the upper membrane surface. Migrated cells were then fixed with 4% paraformaldehyde, stained with 0.1% crystal violet, and counted under a light microscope.

6. Colony Formation Assay

For colony formation assays, organoids, HCT116, and SW480 cells were seeded at a density of 500 cells per well in 3D Matrigel in 24-well plates. Cells were cultured for 5 days, with media replaced every 2 days. Colonies were fixed with 4% paraformaldehyde and counted.

7. Real-Time Quantitative PCR (qPCR)

Total RNA was extracted using TRIzol reagent (Thermo Fisher, USA) according to the manufacturer's instructions. Complementary DNA (cDNA) synthesis was performed using the PrimeScript RT reagent kit (Takara, Japan). Quantitative PCR was conducted using SYBR Green PCR Master Mix (Takara, Japan) on a QuantStudio 5 Real-Time PCR System (Applied Biosystems, USA). Primer sequences were designed based on the guidance of Primer Bank. Relative expression levels were normalized to GAPDH using the 2⁻ΔΔCt method. Each experiment was repeated for independent three times.

8. Western Blotting

Cells were lysed using ice-cold RIPA buffer (Beyotime, China) supplemented with protease and phosphatase inhibitor cocktail (Roche, Basel, Switzerland). Protein concentrations were quantified using the BCA Protein Assay Kit (Thermo Fisher Scientific, Waltham, MA, USA) according to the manufacturer’s instructions. Equal amounts of total protein (20 μg per lane) were separated by SDS–PAGE and transferred onto polyvinylidene fluoride (PVDF) membranes (Millipore, Billerica, MA, USA). Membranes were blocked with 5% non-fat dry milk in TBST for 1 h at room temperature and subsequently incubated overnight at 4°C with the following primary antibodies: anti-IGLC3 (rabbit monoclonal, ab200966, Abcam, Cambridge, UK; 1:1000), anti-BTF3 (rabbit monoclonal, ab203517, Abcam, UK; 1:1000), anti-CD44 (rabbit monoclonal, ab254030, Abcam, UK; 1:1000), anti-Wnt3A (rabbit monoclonal, ab219412, Abcam, UK; 1:1000), and anti-β-actin (mouse monoclonal, ab8226, Abcam, UK; 1:5000), which served as a loading control. After washing with TBST, membranes were incubated with appropriate horseradish peroxidase (HRP)-conjugated secondary antibodies, including goat anti-rabbit IgG-HRP or goat anti-mouse IgG-HRP (Jackson ImmunoResearch, West Grove, PA, USA; 1:5000), for 1 h at room temperature. Protein signals were visualized using an enhanced chemiluminescence (ECL) detection reagent (Bio-Rad, Hercules, CA, USA) and imaged with a ChemiDoc imaging system (Bio-Rad, USA).

9. Immunohistochemistry and Immunofluorescence

Formalin-fixed, paraffin-embedded tissue sections (4 μm) were deparaffinized in xylene and rehydrated through graded ethanol solutions. Antigen retrieval was performed by heating sections in citrate buffer (10 mM, pH 6.0) for 15 min. Endogenous peroxidase activity was quenched using 3% hydrogen peroxide, followed by blocking with 5% bovine serum albumin (BSA). Sections were incubated overnight at 4°C with primary antibodies against BTF3 (rabbit monoclonal, ab203517, Abcam, UK; 1:200) or IGLC3 (rabbit monoclonal, ab200966, Abcam, UK; 1:200). After incubation with HRP-conjugated secondary antibodies (Dako, Denmark), signals were developed using diaminobenzidine (DAB), and nuclei were counterstained with hematoxylin. Images were captured using a light microscope (Olympus, Japan).

For immunofluorescence staining, cells grown on coverslips were fixed with 4% paraformaldehyde for 15 min, permeabilized with 0.1% Triton X-100 for 10 min, and blocked with 5% BSA for 1 h at room temperature. Cells were then incubated overnight at 4°C with the following primary antibodies: anti-SPP1 (rabbit monoclonal, ab214050, Abcam, UK; 1:200), anti-CEA (rabbit monoclonal, ab226144, Abcam, UK; 1:200), anti-CD68 (rabbit monoclonal, ab283654, Abcam, UK; 1:200), anti-Wnt3A (rabbit monoclonal, ab219412, Abcam, UK; 1:200), and anti-BTF3 (rabbit monoclonal, ab203517, Abcam, UK; 1:200). After washing, cells were incubated with species-appropriate fluorophore-conjugated secondary antibodies (Alexa Fluor 488- or 594-labeled goat anti-rabbit IgG, Invitrogen, Carlsbad, CA, USA; 1:500) for 1 h at room temperature in the dark. Nuclei were counterstained with DAPI (Sigma-Aldrich, USA). Images were acquired using a Leica confocal laser scanning microscope (Leica Microsystems, Wetzlar, Germany).

10. ELISA

Levels of TGF-β, CXCL3, SPP1, FN, and IL-1β in culture supernatants were measured using ELISA kits (TGF-β and CXCL3, R&D Systems, USA; FN, Sailofi, China; IL-1β and SPP1, Ciobo, China) according to the manufacturer's protocols.

Supplementary Table I-1

Patient information

| **Characteristics** | **Total (n = 65)** |
| --- | --- |
| Age (years), median (range) | 61 (38–78) |
| Sex, n (%) |  |
| Male | 40 (61.5%) |
| Female | 25 (38.5%) |
| Tumor location, n (%) |  |
| Colon | 48 (73.8%) |
| Rectum | 17 (26.2%) |
| AJCC stage (8th edition), n (%) |  |
| Stage I–II | 32 (49.2%) |
| Stage III–IV | 33 (50.7%) |
| Distant metastasis, n (%) |  |
| Absent (M0) | 30 (46.1%) |
| Present (M1) | 35 (52.8%) |
| Chemotherapy response* (n = 31), n (%) |  |
| Responder | 14 (51.6%) |
| Non-responder | 17 (48.4%) |
| *NA* | *34* |

Supplementary Table I-2

PDO information

| **PDO ID** | **Age** | **Sex** | **Tumor site** | **AJCC stage** | **Metastasis** | **Prior chemotherapy** | **Organoid use** |
| --- | --- | --- | --- | --- | --- | --- | --- |
| PDO-1 | 58 | M | Colon | III | No | No | Macrophage co-culture |
| PDO-2 | 63 | M | Colon | III | No | No | Macrophage co-culture |

Supplementary details:


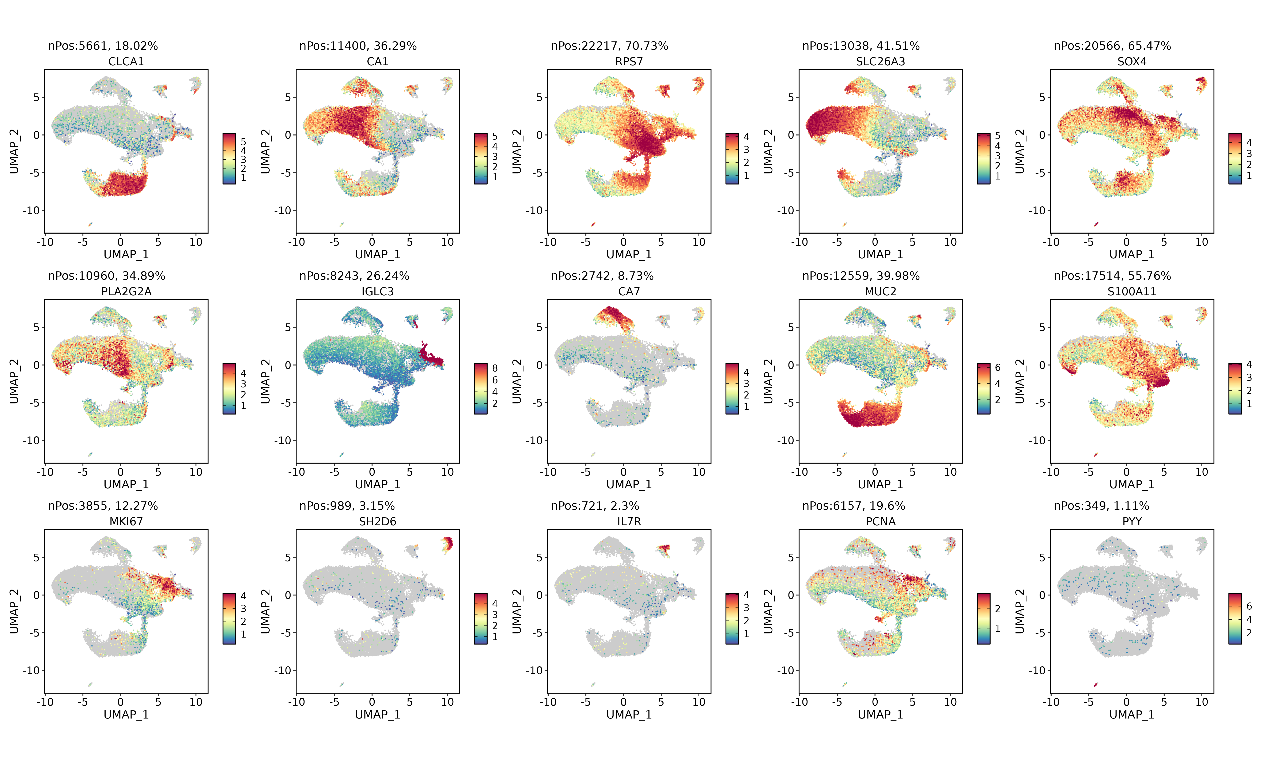


Featureplot of 15 tumor subtypes;


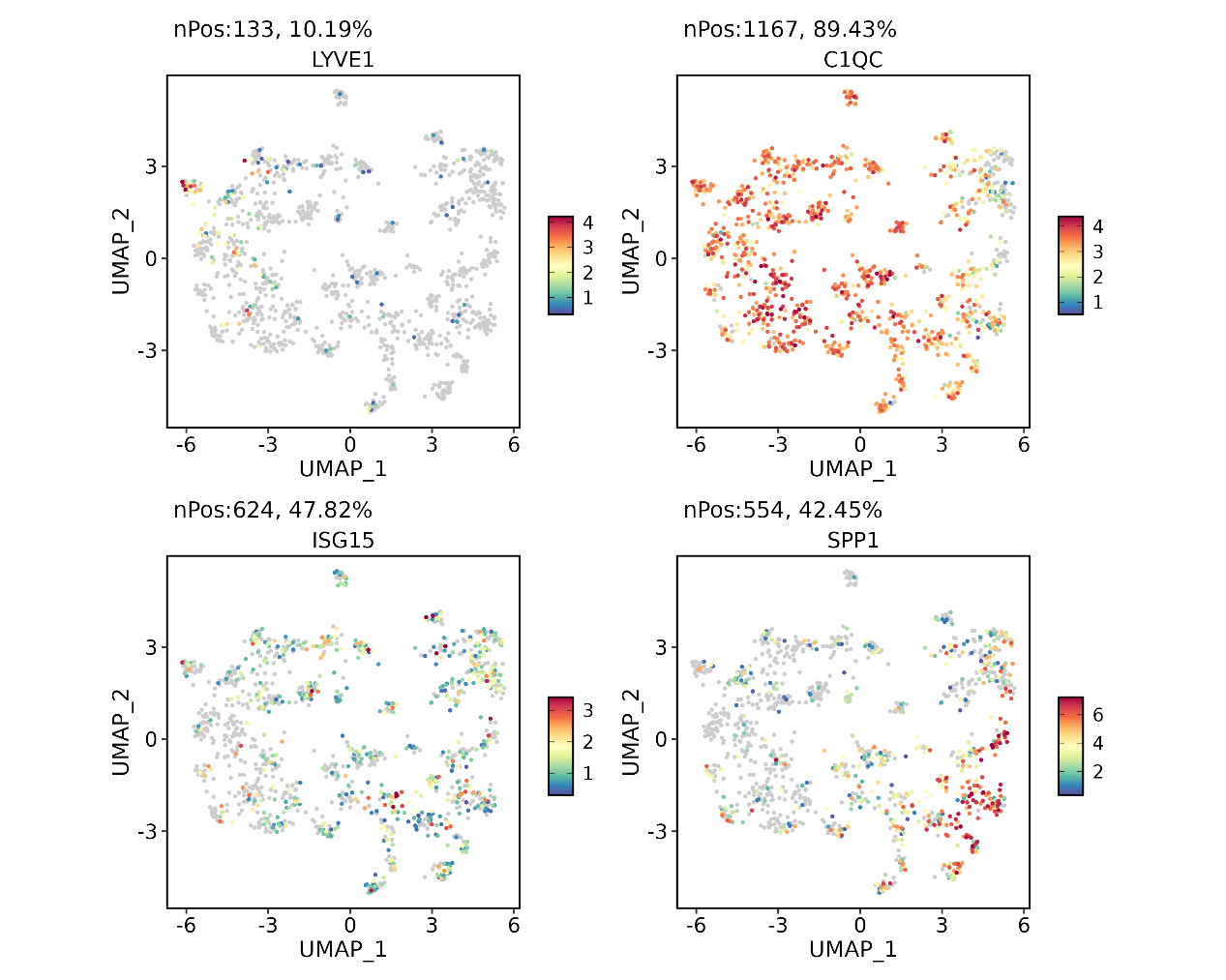


Featuretype of 4 macrophage celltype.
